# Bernard-Soulier syndrome (Hemorrhagiparous thrombocytic dystrophy)

**DOI:** 10.1186/1750-1172-1-46

**Published:** 2006-11-16

**Authors:** François Lanza

**Affiliations:** 1 EFS-Alsace, Strasbourg, France; Inserm, U311, Strasbourg, France; Université Louis Pasteur, Strasbourg, France; 2 INSERM U311/EFS-Alsace, 10 rue Spielmann F, 67065 Strasbourg, France

## Abstract

Bernard-Soulier syndrome (BSS), also known as Hemorrhagiparous thrombocytic dystrophy, is a hereditary bleeding disorder affecting the megakaryocyte/platelet lineage and characterized by bleeding tendency, giant blood platelets and low platelet counts. This syndrome is extremely rare as only ~100 cases have been reported in the literature. Clinical manifestations usually include purpura, epistaxis, menorrhagia, gingival and gastrointestinal bleeding. The syndrome is transmitted as an autosomal recessive trait. The underlying defect is a deficiency or dysfunction of the glycoprotein GPIb-V-IX complex, a platelet-restricted multisubunit receptor required for normal primary hemostasis. The GPIb-V-IX complex binds von Willebrand factor, allowing platelet adhesion and platelet plug formation at sites of vascular injury. Genes coding for the four subunits of the receptor, *GPIBA*, *GPIBB*, *GP5 *and *GP9*, map to chromosomes 17p12, 22q11.2, 3q29, and 3q21, respectively. Defects have been identified in *GPIBA*, *GPIBB*, and *GP9 *but not in *GP5*. Diagnosis is based on a prolonged skin bleeding time, the presence of a small number of very large platelets (macrothrombocytopenia), defective ristocetin-induced platelet agglutination and low or absent expression of the GPIb-V-IX complex. Prothrombin consumption is markedly reduced. The prognosis is usually good with adequate supportive care but severe bleeding episodes can occur with menses, trauma and surgical procedures. Treatment of bleeding or prophylaxis during surgical procedures usually requires platelet transfusion.

## Disease name and synonyms

Bernard-Soulier syndrome (BSS)

Hemorrhagiparous thrombocytic dystrophy

Congenital hemorrhagiparous thrombocytic dystrophy

## Definition and diagnostic criteria

The Bernard-Soulier syndrome (BSS) is an autosomal recessive disease associated with bleeding tendency, giant blood platelets and low platelet counts. The defect is restricted to the megakaryocyte/platelet lineage.

Congenital platelet disorders are often difficult to distinguish on the basis of clinical manifestations and require specialized laboratory tests. Functional analysis of platelet suspensions by aggregometry is needed to differentiate BSS from other rare inherited disorders accompanied by macrothrombocytopenia, such as the May-Hegglin, Sebastian, Fechtner and Epstein's syndromes [1]. A firm diagnosis calls for conjunction of increased bleeding times, macrothrombocytopenia, defective ristocetin-induced agglutination and low or absent levels of platelet GPIb-V-IX (CD42a-d) by flow cytometry.

## Epidemiology

This syndrome is extremely rare as only ~100 cases have been reported in published articles, mostly in the populations of Japan, Europe, and North America. Prevalence has been estimated at less than 1/1,000,000 but is probably higher due to misdiagnosis and underreporting. This low frequency could probably be explained by the fact that the affected genes are very compact being interrupted by only 1 or 2 introns.

## Clinical description

In 1948, Jean Bernard and Jean-Pierre Soulier [2], two French hematologists, described a young male patient who had a severe bleeding defect with a prolonged bleeding time, a low platelet count with very large platelets (macrothrombocytopenia). In view of these defects, they named the disorder "Dystrophie thrombocytaire-hémorragipare congénitale" (Hemorrhagiparous thrombocytic dystrophy). Additional cases presenting with an identical disorder, mostly transmitted as an autosomal recessive trait and often associated with consanguinity, have been subsequently reported [3,4]. In the literature, the disease is now commonly referred to as Bernard-Soulier syndrome (BSS). In most cases, bleeding symptoms manifest rapidly after birth or during early childhood. Clinical manifestations usually include purpura, epistaxis, gingival bleeding and menorrhagia, and more rarely gastrointestinal bleeding and hematuria. Severe bleeding episodes are associated with trauma and surgical procedures such as tonsillectomy, appendectomy, splenectomy, or occur during dental extractions and menses. However, the severity and frequency of bleeding vary between individuals. Bleeding mainly affects mucocutaneous tissues, and major hematomas are very rarely observed.

## Etiology

**Figure 1:**
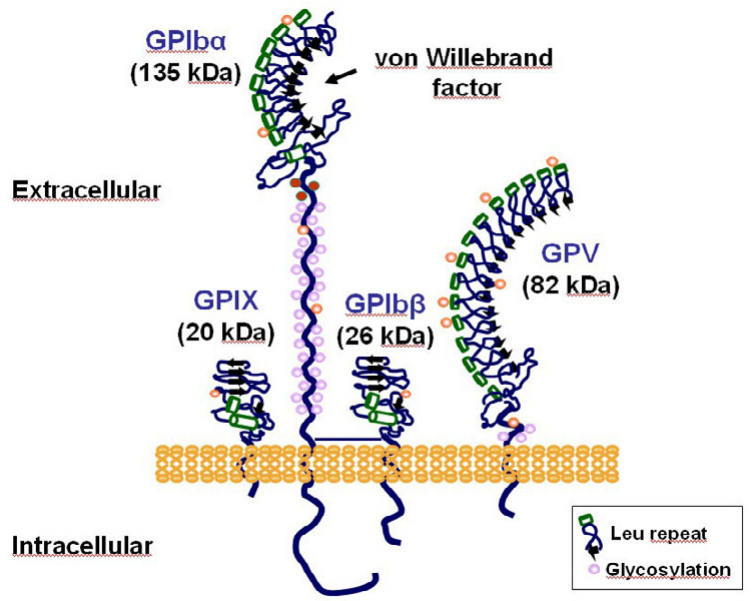
The platelet GPIb-V-IX complex.

Defects in three genes give rise to the typical clinical features and platelet anomalies associated with BSS. This is due to the multisubunit nature of the affected GPIb-V-IX receptor, whose structure is shown in Figure 1. The main function of the GPIb-V-IX complex is to ensure normal primary hemostasis by initiating platelet adhesion at sites of vascular injury [5]. Adhesion is brought by its binding to von Willebrand factor, itself captured from plasma by subendothelial collagen [6]. Four distinct transmembrane proteins, GPIb**α **(MW 135 kDa), GPIb**β **(MW 26 kDa), GPIX (MW 20 kDa) and GPV (MW 82 kDa) assemble to form the functional receptor at the surface of bone marrow megakaryocytes, the precursors of mature circulating platelets [7]. GPIb**α**, GPIb**β**, and GPIX are closely associated and are all required for efficient biosynthesis of the receptor [8]. A lack of a single subunit dramatically decreases surface expression of the whole complex. GPV is more loosely associated and its absence does neither prevent expression, nor von Willebrand factor binding function. The four subunits are separately encoded by genes mapping to chromosomes 17p12 (*GPIBA*), 22q11.2 (*GPIBB*), 3q29(*GP5*) and 3q21 (*GP9*) [9-15]. The four genes belong to the leucine-rich family of proteins and are exclusively expressed in platelets under physiological conditions. They have a simple structure with the coding sequence contained within a single exon, except for *GPIBB *which contains an intron of 10 bases after the start codon.

To date, 47 different genetic defects associated to the BSS have been identified [see Additional file 1]. A Bernard-Soulier registry can also be found at Bernard-Soulier syndrome web site [16]. Defects are due to mutations in *GPIBA *(20 mutations), which is the largest subunit and bears the von Willebrand binding site, in *GPIBB *(16 mutations) and in *GP9 *(11 mutations). The defects can be separated into three major classes: 1) missense mutations or short in-frame deletions which rarely give rise to normal or slightly decreased expression of a dysfunctional receptor or more often to an abnormal/unstable complex with strongly decreased surface expression; 2) nonsense mutations resulting in smaller subunits that typically lack the transmembrane domain; 3) frameshift insertions or deletions leading to a novel polypeptide sequence and often to a premature stop. In very few cases, a single mutation, like in some GPIb**α **and GPIX mutants, affects mainly the function of the receptor, which is at least partially expressed in giant platelets. Most of the defects are unique to a single individual or family. One exception is the *GP9 *Asn45Ser mutation, which has been reported in several families from different countries, probably as a result of a founder effect [17]. The Ala156Val mutation also occurs frequently in the Italian population, with an apparent dominant transmission leading to macrothrombocytopenia in heterozygote individuals [18]. Several mutations combining a BSS phenotype and the DiGeorge/Velo-cardio-facial syndrome (VCF) have been reported. In these cases, one allele carried a mutated *GPIBB *gene, while the other allele had the large deletion of the chromosomal region 22q11.2, containing the *GPIBB *gene, typical of the Di George/VCF defect [19].

Several polymorphisms have been reported especially in the *GPIBA *gene. They include silent mutations, a missense mutation which gives rise to an alloantigen and a variable number of tandem repeat (VNTR) polymorphism [20]. To date, none of these polymorphisms have been linked to anomalies in receptor expression or function.

Rare gain of function mutations of *GPIBA *give rise to platelet-type von Willebrand disease, a clinical entity distinct from BSS. The condition resembles type IIB von Willebrand disease due to clearance of plasma von Willebrand factor by aggregated platelets. Three missense mutations in a disulfide loop flanking the leucine-rich domain and a 9 amino acid deletion in the macroglycopeptide have been reported in these patients [21-24].

## Diagnostic methods

Skin bleeding times in BSS are moderately (5–10 min) to severely (>20 min) prolonged. A constant feature is the presence of a small number of very large platelets with a rounded shape (main volume 11–16 **μ**m^3^; diameter 4–10 **μ**m). The initial laboratory test should therefore include blood cell counts and examination of blood smears. Platelet counts typically range from 20,000 to 100,000/**μ**l. Manual counting is required for an accurate determination as the very large platelets in BSS are often mistaken for lymphocytes in automatic counters. The distinctive abnormality of BSS platelets is an isolated defect in ristocetin-induced agglutination. Unlike the defect in von Willebrand disease, this anomaly is not corrected by the addition of normal plasma. Levels of FVIII-von Willebrand complex are assessed. Aggregation responses to agonists such as ADP or collagen are normal, however decreased responses to thrombin can be observed. A marked defect in prothrombin consumption is constantly observed and may be useful for the diagnosis: it is attributed to a defective binding of FXI due to a lack of GPIb [25], and to a decrease in GPIb-fibrin-dependent thrombin generation [26,27]. Flow cytometry analysis using a panel of specific monoclonal antibodies (CD42 a-d) will confirm this diagnosis. Additional tests in specialized research units may include platelet glycoprotein analysis by SDS-polyacrylamide gel separation and immunoblotting, and finally, study of genetic abnormalities.

## Differential diagnosis

BSS belongs to a heterogeneous group of rare inherited diseases characterized by a reduced number of platelets (thrombocytopenia). A published diagnostic algorithm should facilitate differential diagnosis of BSS [28]. For example, presence of small platelets is typical of Wiscott-Aldrich syndrome and X-linked thrombocytopenia. Giant platelets associated with neutrophil inclusions will orient toward *MYH9*-related diseases (May-Hegglin, Sebastian, Fechtner, and Epstein syndromes). Other parameters will help diagnose macrothrombocytopenia in Di George, Gray platelet, Montreal and Paris-Trousseau syndromes. Finally, some cases of heterozygous BSS can also exhibit macrothrombocytopenia.

BSS is difficult to be distinguished on the basis of clinical manifestations only and has often been misdiagnosed as idiopathic thrombocytopenic purpura (ITP), an immunological disorder, and treated unsuccessfully with steroids or splenectomy.

## Genetic counseling

Genetic counseling should follow the standards established for all autosomal recessive diseases.

## Antenatal diagnosis

Prenatal diagnosis is theoretically feasible when the genetic defect has been identified in a particular kindred. This is probably not justified as the syndrome rarely gives rise to life threatening bleeding. With a good prophylaxis, a fairly normal quality of life can be maintained. In addition, cord blood or chorion villus sampling bear a high risk of bleeding and premature abortion.

## Management

Therapeutic approaches include both general and specific treatment of bleeding. Patients should be warned to avoid traumas, antiplatelet medication such as aspirin, to maintain adequate dental hygiene and to use contraceptive in female at puberty. Treatment of bleeding or prophylaxis during surgical procedures usually requires blood or platelet transfusion with the associated risk of developing antiplatelet alloantibodies. Desmopressin and rFVIIa administration have been shown to shorten the bleeding time in some patients. In rare cases of patients with life-threatening disorders, bone-marrow or umbilical-cord hematopoietic stem cell transplantation may be considered [29]. As a rather simple genetic defect, BSS could be a candidate for future gene replacement therapy using virally transduced megakaryocyte progenitors.

## Prognosis

With a good education, adequate care and prevention of trauma, patients can live a fairly normal life. Nevertheless, possible occurrence of severe bleeding in case of trauma and surgical intervention should always be kept in mind and treated adequately.

## Unresolved questions

To date, no BSS patient has been identified with a mutation of the GPV subunit but existence of such defects cannot be ruled out, and they could potentially result in variant or mild forms of BSS.

As for other platelet deficiencies, it is difficult to correlate genetic defects with propensity to bleed and severity of bleeding.

The molecular and cellular mechanisms responsible for the several platelet defects encountered in BSS are still largely unknown. An explanation for their enlarged platelets and low platelet counts is probably related to defective megakaryocytopoiesis but such studies are difficult to conduct in BSS patients. Additionally, platelet functional studies *in vivo *and evaluation of a putative protection against thrombosis are not amenable in BSS patients. Finally, there is no clear explanation for the abnormal prothrombin consumption in this pathology. Mouse models of BSS are now available with deletions of the GPIb**α **or GPIb**β **subunit which should help answering these remaining questions.

## Supplementary Material

Additional file 1 Genetic defects in Bernard-Soulier syndrome. This is a table that represents the genetic defects in Bernard-Soulier syndrome and corresponding references. Click here for file
