# Supplementary material for: Bernard-Soulier syndrome (Hemorrhagiparous thrombocytic dystrophy)
Source: Orphanet J Rare Dis. 2006 Nov 16;1:46. doi: 10.1186/1750-1172-1-46 (PMC1660532; doi:10.1186/1750-1172-1-46)
Supplement: Additional file 1 — Genetic defects in Bernard-Soulier syndrome. This is a table that represents the genetic defects in Bernard-Soulier syndrome and corresponding references. [file 1750-1172-1-46-S1.doc]

Genetic defects in Bernard-Soulier syndrome

| **Gene** | **Missense mutations or short deletions** | **Nonsense mutations** | **Frameshift->Stop** | **Other** | **References** |
| --- | --- | --- | --- | --- | --- |
| *GPIBA* | Tyr54->Asp (het)(macrothrombocytopenia) |  |  |  | Kunishima S *et al*, 2006 [30] |
|  | Leu57->Phe |  |  |  | Miller HL *et al*, 1992 [31] |
|  | Cys65->Arg |  |  |  | Kenny D *et al*, 1998 [32] |
|  | Leu123->Pro |  |  |  | Proulle V *et al*, unpublished |
|  | Leu126->Phe |  |  |  | Furihata K *et al*, unpublished |
|  | Leu129->Pro |  |  |  | Li C *et al*, 1995 [33]; Antonnuci JV *et al*, 2000 [34]; Koskela S *et al*, 1999 [35] |
|  | Ala156->Val |  |  |  | Ware J *et al*, 1993 [36]; Lanza *et al*, unpublished; Margaglione M *et al*, 1999 [37]; Savoia A *et al*, 2001 [18] |
|  | **∆**Leu179 |  |  |  | de la Salle C *et al*, 1995 [38]; Ulsemer P *et al*, 2000 [39]; Ulsemer P *et al*, 2001 [8] |
|  | **∆**169-180/Glu81->Lys |  |  |  | Margaglione M *et al*, 1999 [37] |
|  | Cys 209->Ser |  |  |  | Simsek S *et al*, 1994 [40]; Gonzalez-Manchon C *et al*, 2001 [41]; Lanza *et al*, unpublished; |
|  |  | Trp343->Stop |  |  | Ware J *et al*, 1990 [42] |
|  |  | Ser444->Stop |  |  | Kunishima S *et al*, 1994 [43] |
|  |  | Trp498->Stop |  |  | Holmberg L *et al*, 1997 [44]; Kenny D *et al*, 1998 [32] |
|  |  |  | Lys19->Arg + 2AA->Stop |  | Li C *et al*, 1996 [45] |
|  |  |  | **∆**Ser39Glu40 + 51AA->Stop |  | Afshar-Kharghan V *et al*, 2000 [46] |
|  |  |  | Arg76->Leu + 19AA->Stop |  | Simsek S *et al*, 1994 [47] |
|  |  |  | Val295->Gly + 39AA->Stop |  | Kanaji T *et al*, 1997 [48] |
|  |  |  | Ser444->Ile + 11AA-Stop (het) |  | Kanaji T *et al*, 1997 [48]; Gonzalez-Manchon C *et al*, 2001 [41] |
|  |  |  | Thr452->Pro + 58AA->Stop |  | Noda M *et al*, 1995 [49];1996 [50]; Mitsui T *et al*, 1998 [51]; Kanaji T *et al*, 1997 [48] |
|  |  |  | Tyr492->Cys + 80AA->Stop |  | Afshar-Kharghan V and Lopez JA, 1997 [52]; Kenny D *et al*, 1997 [53]; Koskela S *et al*, 1999 [35] |
| *GPIBB* | Cys5->Tyr |  |  |  | Gonzalez-Manchon C *et al*, 2003 [54] |
|  | Arg17->Cys(Giant platelets) |  |  |  | Kunishima S *et al*, 2001 [55] |
|  | Pro29->Leu (het) |  |  |  | Hillmann A *et al*, 2002 [56] |
|  | Asn64->Thr |  |  |  | Strassel C *et al*, 2003 [57] |
|  | Pro74->Arg |  |  |  | Kunishima S *et al*, 2000 [58] |
|  | Tyr88->Cys |  |  |  | Kurokawa Y et al, 2001 [59] |
|  | Tyr88->Cys +Ala108-Pro (het) (Giant platelets) |  |  |  | Kunishima S *et al*, 1997 [60] |
|  | Pro96->Ser + Di George-VCF |  |  |  | Tang J *et al*, 2004 [61] |
|  | Cys122->Ser (het) |  |  |  | Kunishima S *et al*, 2004 [62] |
|  |  | Trp21->Stop |  |  | Moran N *et al*, 2000 [63] |
|  |  | Cys116->Stop |  |  | de la Salle C *et al*, 1997 [64] |
|  |  | Trp123->Stop |  |  | Kunishima S *et al*, 2002 [65], 2004 [62] |
|  |  |  | Gly81->Ala + 86AA->Stop +Di George-VCF |  | Kenny D *et al*, 1999 [66] |
|  |  |  | **∆**13bp Signal sequence + 22AA->Stop |  | Watanabe R *et al*, 2003 [67]; Strassel *et al*, 2004 [68] |
|  |  |  | Ala131->Gly +153AA->Stop |  | Strassel *et al*, 2006 [69] |
|  |  |  |  | Promoter Mutation GATA + Di George-VCF | Budarf ML *et al*, 1995 [19]; Ludlow LB *et al*, 1996 [70] |
| GP9 | Leu(-10)->Pro |  |  |  | Lanza F *et al*, 2002 [71] |
|  | Cys8->Arg |  |  |  | Rivera CE *et al*, 2001 [72] |
|  | Asp21->Gly (het) |  |  |  | Wright SD *et al*, 1993 [73] |
|  | Leu40->Pro |  |  |  | Noris P *et al*, 1998 [74] |
|  | Asn45->Ser (homo and het) |  |  |  | Wright SD *et al*, 1993 [73]; Clemetson JM *et al*, 1994 [75]; Donner M *et al*, 1996 [76]; de la Salle *et al*, 1997 [64]; Koskela S *et al*, 1999 [77]; Vanhoorelbeke K *et al*, 2001 [78]; Sachs UJ et al, 2003 [79]; Drouin J *et al*, 2005 [80]; Lanza F *et al*, unpublished ; Liang *et al*, 2005 [17] |
|  | Phe55->Ser |  |  |  | Noris P *et al*, 1997 [74]; Suzuki K *et al*, 1997 [81]; Suzuki K *et al*, 1999 [82] |
|  | Cys73->Tyr |  |  |  | Noda M *et al*, 1996 [50] |
|  | Asn86->Ala DArg87-Pro89 (het) |  |  |  | Drouin J *et al*, 2005 [80] |
|  | Cys97->Tyr |  |  |  | Kunishima S *et al*, 1999 [83] |
|  | Ala140->Thr |  |  |  | Wang Z *et al*, 2004 [84]; Garner *et al*, 2006 [85] |
|  |  | Trp127->Stop |  |  | Noda M *et al*, 1995 [49]; Iwanaga M *et al*, 1998 [85] |

**Δ**: deletion

Amino acids (**AA**) are in three letter code

**(->)** change from wild type to mutated AA sequence

Numbering is based on the mature sequence (GPIX Leu(-10)->Pro is located in the signal peptide)

**Giant platelets:** variant phenotype not a BSS

**(het)** compound heterozygote

**Di George-VCF:** Di George (Velo-Cardio-Facial syndrome) deletion of chromosome region 22q11.2 on the second allele
